# Editorial

**Published:** 1914-03

**Authors:** 


### EDITORIAL

The Business Office of the *Journal-Record* is 23 West Alabama Street.

The Editorial Office is 1014-15 Atlanta National Bank Building.

Address all Business Communications to *Journal-Record of Medicine,* 23 West Alabama Street.

Make remittances payable to *The Journal-Record of Medicine.*

On matters pertaining to the Editorial and Original Communications, address Edgar G. Ballenger, M. D., Atlanta, Ga.

Reprints of Original Articles will be furnished at cost price. Requests for the same should always be made in THE MANUSCRIPT.

We will present, postpaid, on request to each contributor of an original article, twenty (20) marked copies of *The Journal-Record of Medicine* containing such article.

### GRADY HOSPITAL.

The City of Atlanta is coming into its heritage and doesn't quite know what to do with it. It has had its growing pains and attended to them as best it could and sometimes just endured them because it thought there was no remedy, but now in its lusty majority, it is stretching its limbs, swelling its powerful muscles, getting to man's estate and beginning to look ahead.

Among other things it is seen that the care of the sick is a matter of civic economics that can not be permitted to go any longer by hit or miss methods to whatever end happens to be convenient. It realizes that science should have control and have opportunity to control disease.

The present hospital facilities are inadeqate to the number of sick seeking them both in the private and the public institutions, and besides this, no small hospital can have the means for adequate laboratory diagnosis and special treatments unless it is far more richly endowed than any of the sanitoriums now in existence are likely to be. No unfavorable criticism is intended upon what has been done. Every man interested in the progress of medicine has done his best and Atlanta is in the forefront of cities as to its professional skill and philanthropic activities.

What is needed is a large Hospital embracing every known facility for diagnosis and care of the sick and with room for expansion as the future demands it. The city is in a fair way to issue bonds to provide such a hospital, to extend the Grady Hospital to meet the demands of the people and to lend every possible opportunity to scientific men in their work.

With an institution at least quadruple the size of the present one, permanent skilled service of internes could be secured that would give the laboratory the attention almost impossilble to secure at present and it would enable workers to extend their labors in every field as far as the world's knowledge would show the way.

For many things, there is scarcely any space in Grady at present. The great burden of venereal diseases, for example, receives only minor attention because of lack of space. Every where there is the stress of crowding that the Administration can not relieve. Patients feel it and their progress toward recovery is hindered by it. Sick people outside decline to go to the Hospital because of the crowding and everyone suffers because of it. The relief must come from the citizens and the public purse.

The plan should include response to present needs and should embrace as well plans for an Institution where every illness could be carefully studied and adequately treated. It should be so arranged as to permit of expert investigation and high grade teaching. This part of the South needs such an institution every day and our men are constantly going to Baltimore, Rochester and eastern cities for instruction and experience that is to be had right in Atlanta if proper opportunity is given for the work. We can not too earnestly urge upon all our citizens to work for the general good that will come with the development of the great Grady Hospital.

R. R. D.

---

### MALARIA.

Malaria has been with us for so many years, we almost feel we can not get on without it and that anyway, it is somewhat of a routine matter coming and going without our control, and which we have little more to do with than to endure.

Familiarity has bred a contempt for it that blinds us to its consequences. We know it does kill a certain number of its sufferers, but the loss is not great as compared with other diseases. We are likely to think that many of the deaths ascribed to "malaria" are probably something else than results of this disease and that the disease itself is as indefinite as its name.

We overlook the fact that the disease causes great economic loss through the disabilities associated with it, such as neurasthenia, lack of nutrition and development in the young and impairment of efficiency in the adult. If we knew what it costs every community in this way, we would be sure that we could not afford to buy it as a luxury and we would begin to see that we can not afford to permit its existence as an unnecessary burden.

Dr. Graham E. Henson of Jacksonville, Fla., is doing splendid work these days in taking up the devastations of Malaria and making them clear to the world and at the same time, showing the uselessness of all the suffering.

Malaria, despite its misleading name, is a definite disease depending upon a readily recognized organism in the patient's blood for its existence and running courses that have been learned and may be easily followed in its life cycles.

He is advising us to extend our work in the prevention of the disease by taking up the human host and caring for him while he entertains the communicable poison. The work on the Isthmus and at other similar places in the extermination of the mosquito is admirable, but it does only a part and it leads many of us who are not investigators to overlook the fact that the mosquito is harmless, unless it has the organism to carry to the human it bites. The mosquito can get the organism only from some human host already suffering with the disease in the communicable form. It developes the germ to a certain point in its peculiar cycle and then injects it into the blood of the next human who will play host and have the disease.

It has been clearly demonstrated that men can act as hosts of malaria without suffering greatly themselves and that they can infect a whole community by transmitting the disease to the neighboring mosquitoes. It has been shown with equal precision that the disease can be cured by proper doses of quinine.

We have then these factors: disease that invariably shows its organism in the blood of patients when specimens are taken at proper times. The development of the organism so that a mosquito that bites the patient will have the organism in its stomach. The mosquito will carry this further evolved organism to the next man it bites and thus complete the disease cycle and spread the infection.

It is evident, therefore, that the blood of every man suspected of having malaria should be examined. This may readily be done with the laboratory assistance the State is prepared to give. Then the patient should be isolated from all chance of infecting mosquitoes and given such treatment as will cure him.

Small communities have been entirely freed from malaria in a few months even where they have not been able to do the extensive drainage necessary to destroying the mosquitoes. They have simply killed off the malarial organisms by preventing their developing into the communicable stage while they were in the human hosts.

We urge this upon our readers even to the extent of making malaria a quarantine disease. The results of careful diagnosis, prophylaxis and treatment would be brilliant enough to satisfy every grumbler and to protect every community from great financial loss.

Let us awaken to the importance of a situation in which we are chronic sufferers with a disease that can be completely removed if we make the careful study Henson outlines and apply the simple treatment he advises. Isolate and cure the human patients and the mosquitoes will cease to carry malaria.

